# Adolescence and innovation in the European Upper Palaeolithic

**DOI:** 10.1017/ehs.2020.37

**Published:** 2020-06-22

**Authors:** April Nowell, Jennifer C. French

**Affiliations:** 1 Department of Anthropology, University of Victoria, PO Box 1700 STN CSC, Victoria, BC, Canada V8W 2Y2; 2 University College London, Institute of Archaeology, 31–34 Gordon Square, London WC1H 0PY, UK

**Keywords:** Adolescence, innovation, Upper Palaeolithic, hunter–gatherers, demography

## Abstract

Childhood and adolescence are two stages of development that are unique to the human life course. While childhood in the Pleistocene has received considerable attention in recent years, adolescence during the same period remains an understudied area of research. Yet it is during adolescence that key social, physical and cognitive milestones are reached. Thus, through studying adolescents, there is enormous potential for improving our understanding of Upper Palaeolithic lifeways more broadly. The reason for the dearth of these types of studies may be the perceived methodological difficulty of identifying adolescents in the archaeological record. In many ways, it is easier to distinguish children (*sensu lato*) from adults based on size, developmental age and associated artefacts. Adolescents, however, are often seen as more ambiguous, more liminal. Working within an evolutionary framework and using a definition of adolescence rooted in biology, we draw on psychology, ethnography and palaeodemography to develop a model of what it might have meant to be a ‘teenager’ in the European Upper Palaeolithic**.** Citing the biological, social and cognitive changes that occur during this life stage, we propose an important role of teenagers in the origins and spread of new ideas and innovations throughout the Late Pleistocene.

## Graphical abstract

**Figure:**
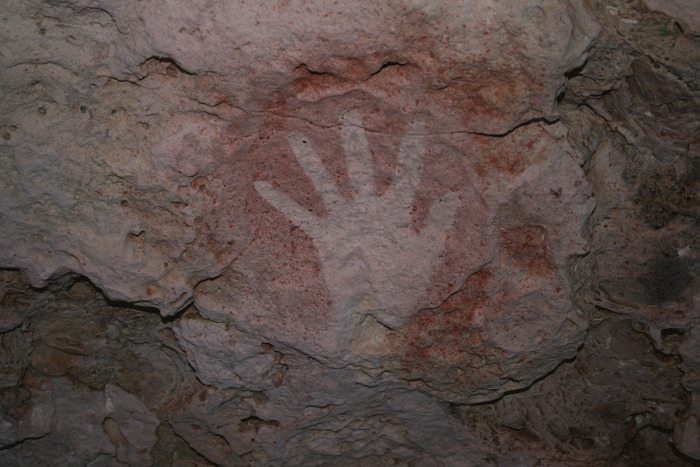

**Media summary:** Teenagers played an important role in the origin and spread of new ideas and innovations throughout the Late Pleistocene.

## Introduction

Childhood and adolescence are two stages of development that are unique to the human life course. While childhood in the Pleistocene has received considerable attention in recent years (e.g. Fischer, 1990; Grimm, 2000; Roveland, 2000; Sharpe & Van Gelder, 2004, 2006; Shea 2006; Stapert, 2007; Nowell and White, 2010; Bahn, 2015; Finlay, 2015; Nowell, 2015a, b, 2016, 2020, 2021; Van Gelder, 2015a,b; Langley, 2018; Langley & Lister, 2018; Riede et al., 2018), adolescence during the same period remains an understudied area of research. The reason for the dearth of studies of adolescence may be the perceived methodological difficulty of identifying adolescents in the archaeological record. In some sense, it feels more straightforward to distinguish children from adults based on size, developmental age and associated artefacts. Adolescents, in contrast, are often seen as more ambiguous, more liminal – *when does a ‘teen’ become an adult?*

Working within an evolutionary framework and using a definition of adolescence rooted in biology, we draw on psychology, ethnography and palaeodemography to develop a model of what it might have meant to be a ‘teenager’ in the European Upper Palaeolithic (ca. 40,000–12,000 years ago). Our review indicates that adolescence is marked by intensive biological, cognitive and psycho-social changes that have prompted psychologists to describe adolescence as a time of increased creativity, flexibility, exploration and risk taking. We link these adolescent changes to the spread of new ideas and innovations, proposing that teenagers in Upper Palaeolithic hunter–gatherer communities were crucial to the rapid development and dissemination of the new forms of material culture and social interactions that characterised this period of prehistory (Pettitt, 2014).

## Defining adolescence: biological, social and cognitive features

### Biology

Adolescence as a human life history stage is defined as the period from the onset of the adolescent growth spurt to its cessation (Bogin, 2003, 2009). The adolescent growth spurt is a uniquely human phenomenon. During this time individuals gain 50% of their weight and 20% of their final adult stature and it encompasses sexual maturation, i.e. puberty. While a boy is fertile before he develops any secondary sexual characteristics (e.g. enlargement of larynx and deepening of voice, growth of facial and body hair including pubic hair, increased stature), a girl ‘develops nearly all of her secondary sexual characteristics [e.g. breasts, underarm and pubic hair, increased stature] to their fullest extent before acquiring her fertility’ (Lancaster, 1986: 21). In addition, girls only achieve adult pelvic dimensions at the very end of puberty. What this difference in patterning suggests is that, evolutionarily, there has been selection for delaying fertility in human females until they have had time to function socially as adults (Lancaster, 1986). With the onset of puberty, another noticeable characteristic of adolescence emerges – a significant shift in sleep patterns. Alterations to circadian and homeostatic systems lead to increasingly later releases of melatonin throughout puberty. This means that teens feel the need to sleep later and wake later than others do (Galván, 2020).

The order of the biological changes that mark adolescence is universal, but the timing and speed of change varies between the sexes and is influenced by an individual's social environment (see discussion below). Definitions of adolescence based on these biological changes suggest that in twentieth- and twenty-first-century contexts this life stage begins at around age 10 (Sawyer et al., 2018). The end of adolescence is less clearly defined biologically. The cessation of the adolescent growth spurt (~16–17 years) provides a clear biological and developmental marker for the end of puberty, but whether this corresponds with the end of adolescence is debated, and other developmental features that characterise adolescence suggest a later end. For example, Roenneberg et al. (2004) propose that another abrupt change in sleeping patterns at age 20 marks the biological end of adolescence.

### Social

Adolescence is universally recognised as a distinct social, as well as biological, stage of development (Schlegel & Barry, 1991). Biological age, which is estimated from biological changes in the body, is not necessarily the same as social age, which is defined as ‘the culturally constructed norms of appropriate behaviour and status of individuals within an age category’ (Halcrow & Tayles, 2008: 192). However, these two types of age are interrelated as social and environmental factors such as degree of physical activity, access to nutritious food, familial stress, life expectancy and socioeconomic stress can impact the onset and duration of puberty (Halcrow & Tayles, 2008; Nabi et al., 2014; Lewis et al., 2016). Furthermore, the development of secondary sexual characteristics may signal readiness to take on behaviours associated with specific social age categories (see, for example, Lewis et al., 2016).

The social aspect of adolescence is no less important than the biological in terms of defining this life stage. As part of the recognition of having reached a new life stage, adolescents are treated differently from both younger children and adults. These differences usually relate to preparing adolescents for their future economic and family roles and responsibilities, and accordingly, vary between the sexes (Schlegel & Barry, 1991, pp. 33–35). Social definitions of adolescence are more variable than those based on biology but they both share a difficulty in defining the end of the adolescence phase. In recent industrialised contexts, Sawyer et al. (2018) propose extending the end of adolescence to 24 years (from earlier definitions of 19 years) to reflect social trends including a later age at marriage, longer periods of education and delayed economic independence. We need to remember, however, that while almost all cultures recognised adolescence as a distinct life-stage, ‘the modern adolescent is not the modal adolescent’ (Schlegel & Hewlett, 2011: 287); who is considered an ‘adolescent’ and the roles and responsibilities of these individuals are culturally variable. In this regard, the social investment theory suggests that adolescents transition to adulthood and mature more quickly in cultures where they assume adult responsibilities at an earlier age (Bleidorn et al., 2013 and references therein).

### Cognitive

As Arain et al. (2013: 451) observe, ‘adolescence is one of the most dynamic events of human growth and development, second only to infancy in terms of the rate of developmental changes that occur in the brain’. While there is individual variation in adolescent brain maturation, in broad strokes, throughout puberty, cortical grey matter decreases as a result of synaptic pruning while white matter gradually increases possibly as a result of increased myelination (the process by which white matter tracts are covered with a fatty coating to conduct signals more efficiently; Khundrakpam et al., 2016; O'Rourke et al., 2020). Thus during adolescence, in what has been described as a ‘use it or lose it’ process (O'Rourke et al. 2020), pruning removes excess neurons and synaptic connections that are no longer needed by the brain in order to improve the efficiency of neuronal transmission (Khundrakpam et al., 2016). Pruning represents ‘the behavioural, and ultimately, the physiological suppression of competing, irrelevant behaviours’ (Casey et al., 2000: 246). This process reflects the importance of both biology and environmental experience on an adolescent's brain maturation (O'Rourke et al., 2020).

Different regions of the brain mature at different rates. For example, during adolescence, there are significant changes in the subcortical limbic system governing emotion and mood. However, the prefrontal cortex (PFC), governing executive functions such as attention, inhibition, cognitive flexibility and the ability to plan and think through the consequences of an action, continues to develop throughout adolescence. In fact, the PFC is actually the last region of the brain to fully mature at around age 25 (Burnett & Blakemore, 2009; Khundrakpam et al., 2016; O'Rourke et al., 2020). This diversity in maturation rates can lead to greater risk taking, impulsivity and emotionally driven decision making in teens without the necessary checks and balances to constrain that behaviour being fully available.

Social cognition in teens is equally complex. Activation of the amygdala during pubertal development leads to an increased ability to read emotions in the faces of others (O'Rourke et al., 2020) and in taking on other emotional perspectives – what Burnett and Blakemore (2009: 52) describe as the ability to ‘step into someone else's shoes’. During puberty, gonadal hormones involved in reorganising neural circuitry in both males and females lead to greater motivation for sexual and romantic partners (O'Rourke et al., 2020). At the same time, fMRI studies demonstrate that teens activate different regions of the brain than adults when processing situations that require social understanding. This suggests that, over time, understanding social situations becomes more automatic and less laborious, as teens garner more life experience and as neural circuitry is fine-tuned (Burnett & Blakemore, 2009; Blakemore, 2018). As Burnett and Blakemore (2009: 54–55) note, ‘an unexplored implication of this could be that the period of life when arMPFC [anterior rostral medial prefrontal cortex] and other social brain regions are still developing – the teens and early 20s – might be a period of particular open mindedness to new ideas and different types of people’. Similarly, O'Rourke et al. (2020) argue that the unique plasticity of the adolescent brain renders them more open to acquiring new skills. This plasticity also creates an opportunity for learning and motivation relevant to romantic and sexual behaviour (ibid.).

## Identifying adolescents in the Upper Palaeolithic archaeological record

The Palaeolithic has long been recognised as highly susceptible to androcentrism (Conkey & Spector, 1984: 6). The continued dominance of the ‘Man the Hunter’ paradigm (Conkey, 2013; Zihlman, 2013) means that an (often implicit) assumption of the archaeological record as a product of men and their behaviours persists, with direct ‘proof’ of the presence of women and children – including adolescents – required before they can be included in interpretations. As numerous scholars in the fields of gender archaeology and the archaeology of childhood have argued, this position is illogical and hinders the understanding of both past societies and the resultant archaeological record (e.g. Conkey & Gero, 1997; Kamp, 2001). Combined, children and adolescents would have comprised approximately half of past populations (Chamberlain, 2000); no special pleading is required to incorporate adolescents in our consideration of Late Pleistocene lifeways. Direct evidence does, however, provide an excellent starting point for examining teenage life in the Upper Palaeolithic.

Crucially, many (but not all) of the biological changes described above that define adolescence leave traces on the skeleton. Shapland and Lewis (2013, 2014; see also Lewis et al., 2016) have identified a number of dental and skeletal markers for identifying adolescents including: (a) age at death (e.g. calcification of permanent mandibular dentition; fusion of the proximal humerus, proximal and distal tibia and femur; extension of the epiphysis of the ischio-pubic ramus; flaked epiphysis at the medial clavicle, fusion of the ischial epiphysis, sacrum and vertebral annular rings); (b) pubertal stage (based on markers visible on the mandibular canine root, the cervical vertebrae, bones of the hand, elbow and wrist, as well as maturation of the iliac crest); and (c) onset of menarche (e.g. presence of an ossified iliac crest epiphyses or fusion (of any part) of the crest; fusion of the distal phalanx of the second finger where possible or in lieu of this, fusion of any distal hand phalanges).

A survey of the literature identified 50–80 Upper Palaeolithic fossil individuals who can be considered adolescents, the majority of whom come from Europe (Table 1). Inconsistency between researchers in terms used and how individuals are reported make it difficult to ascertain the exact number of adolescents in the fossil record. Authors often employ categories such as ‘infant’, ‘child’, ‘sub-adult’ and ‘non-adult’ vs. ‘adult’ but they rarely, if ever, use the term adolescent. When available, exact age-at-death estimates permit the more reliable identification of adolescents, but the variety in chronological definitions of adolescence (both social and biological) described above supports casting a relatively wide net in our categorisation of ‘adolescents’ in the Upper Palaeolithic to include individuals aged from ~10 years at death into their early 20s. Most of the Upper Palaeolithic adolescents listed in Table 1 come from burial contexts and are often fragmentary. Nonetheless, researchers can look for patterns in such factors as age, sex, grave goods, body positions and pathologies to draw some preliminary conclusions about adolescence in the Upper Palaeolithic (Box 1).

**Table 1.** Upper Palaeolithic adolescent fossil individuals

Individual	Chrono-cultural attribution	Country	Context	Age estimate (years at death)	Sex	Associated ^14^C dates (uncalibrated BP)/estimated absolute date	References
Arene Candide I (‘Il Principe’)	G	Italy	Single burial Primary deposition	12–18	M	23 440 ± 190 (OxA-10700)	Giacobini, 2007; Henry-Gambier, 2008b; Pettitt et al., 2003
Abri Dubalen (Brassempouy)	A	France	Isolated element (tooth)	12 ± 30 months	?	31,520 ± 360 (Gif/LSM-10657)	Gambier, 2000
Arene Candide 10	EG	Italy	Primary/disturbed primary burial	20–29	M	11,605 ± 445 (GX-16960-A)	Bietti and Molari, 1994; Gazzoni and Fontana, 2011; Sparacello et al., 2018
Arene Candide 13	EG	Italy	Secondary deposition	20–22	?		Formicola, 2007; Gazzoni and Fontana, 2011; Sparacello et al., 2018
Arene Candide 16/‘Zona A’	EG	Italy	Primary/disturbed primary burial	12–17	M	10,810 ± 65	Sparacello et al., 2018
Baousso da Torre 2/Balzo della Torre 2 (BT 3)	G	Italy	Single burial	20–29	M		Formicola and Holt, 2015; Gazzoni and Fontana, 2011; Giacobini, 2006; Villotte et al., 2011
Baousso da Torre 3/Balzo della Torre 3 (BT 3)	G	Italy	Primary deposition Single burial	10–18	M		Formicola and Holt, 2015; Giacobini, 2007; Villotte et al., 2011, 2017; Villotte, 2018
Barma Grande 3 (BG 3)	G	Italy	Primary deposition Multiple burial (with BG 2 and BG 4)	12–13	F?	14,990 ± 80 (Beta-6 3510/CMAS-7641)	Formicola, 1988; Formicola and Holt, 2015; Gazzoni and Fontana, 2011; Giacobini, 2007; Henry-Gambier, 2008; Henry-Gambier, 2008b; Onoratini et al., 2012
Barma Grande 4 (BG 4)	G	Italy	Primary deposition Multiple burial (with BG 2 and BG 3)	12–15	F?	14,990 ± 80 (Beta-6 3510/CMAS-7641)	Formicola, 1988; Formicola and Holt, 2015; Henry-Gambier, 2008a, b; Gazzoni and Fontana, 2011; Giacobini, 2007; Onoratini et al., 2012
Caviglione 1/Barma del Caviglione 1/‘Dame du Cavillon’	G	Italy	Single burial	Young adult	F	22,400–26,700 (calibrated)	Chevalier, 2019; Formicola and Holt, 2015; Gazzoni and Fontana, 2011; Giacobini, 2007
Cussac 1 (Locus 3)	G	France	Co-mingled remains of multiple individuals of which at least one is adolescent Primary deposition	Late adolescent	?	25,120 ± 120 (Beta 156643)	Aujoulat et al., 2002; Formicola, 2007; Peigneaux et al., 2019
Dolni Vĕstonice 13 (DV II)	G	Czech Republic	Multiple burial (DV 13, DV 14, DV 15)	17–19	M	27,660 ± 80 (GrN-13692); 26,640 ± 110 (GrN-14831); 24,000 ± 900 (ISGS-1616); 24 970 ± 920 (ISGS-1617)	Alt et al., 1997; Formicola, 2007; Formicola et al., 2001; Hillson et al., 2006; Mittnik et al., 2016; Trinkaus et al., 2001
Dolni Vĕstonice 14 (DV II)	G	Czech Republic	Multiple burial (DV 13, DV 14, DV 15)	16–17	M		
Dolni Vĕstonice 15 (DV II)	G	Czech Republic	Multiple burial (DV 13, DV 14, DV 15)	20	M?		
Dolni Vĕstonice 17	G	Czech Republic		6–10		27–25,000	Hillson et al., 2006; Svoboda, 2007; Trinkaus et al., 2000, 2010
Dolni Vĕstonice 31	G	Czech Republic	Isolated element	21–25	?		
Dolni Vĕstonice 32	G	Czech Republic	Isolated element	21–25	?		
Dolni Vĕstonice 33	G	Czech Republic	Isolated element	Mid-adolescent	?		
Dolni Vĕstonice 37	G	Czech Republic	Isolated element	16–20	?		
Dolni Vĕstonice 64	G	Czech Republic	Isolated element	<18	?		
Dolni Vĕstonice 9	G	Czech Republic	Isolated element	21–25	?		
Eshkaft-e Gavi A11-2098	B	Iran	Isolated element	>12–13	?	>28,000–18,000	Scott and Marean, 2009
Eshkaft-e Gavi A11-2099	B	Iran	Isolated element	>14–16	?	>28,000–18,000	
Eshkaft-e Gavi A11-2208	B	Iran	Isolated element	>13–16	?	>28,000–18,000	
Grotta dei Fanciulli 5 (Enfants)	G	Italy	Primary deposition Burial	Adolescent	M		Formicola and Holt, 2015; Gazzoni and Fontana, 2011; Giacobini, 2007; Henry-Gambier, 2008a; Mallegni and Parenti, 1972–1973; Riel-Salvatore and Gravel-Miguel, 2013
Grotta dei Fanciulli 6 (Enfants)	G	Italy	Primary deposition Burial	12–15	M		
Grotte des Hyénes 1 (Brassempouy)	A	France	Isolated elements	10 ± 30 months	?	31,820 ± 550 BP ± 510 (Gif 8568)	Gambier, 2000
Grotte des Hyénes 3 (Brassempouy)	A	France	Isolated elements	9 ± 24 months	?	31940 ± 160 (Gif/LSM-11035)	Gambier, 2000
Kostenki 18 (Khvoikovskaia)	G	Russia	Primary deposition Single burial	9–10	?	23,440 ± 150 (OxA-X 2666-53)	Henry-Gambier, 2008a, b; Sinitsyn, 2004; Reynolds et al., 2017
Krems-Wachtberg	G	Austria	Isolated element	‘Adolescent’	?	28,300 ± 270 (VERA-3932); 27,420 + 240/−230 (VERA-3933); 27,190 + 230/−220 (VERA-3934); 27,230 + 230/− 220 (VERA-4533);28,000 + 250/− 240 (VERA-4534); 26,800 ± 220 (VERA-5196)	Simon et al., 2014
La Chaise de Vouthon	A	France	Isolated element	8–10	?		Gambier, 2000
La Quina (Qui-A 2)	A	France	Isolated element	12–15	?	31,170 ± 350 (GRO-1493); 30,760 ± 490 (GRO-1489); 32 650 ± 850 (OxA-6147 (Ly-256)); 33 290 ± 330 (OxA-15054)	Verna et al., 2012
La Quina (Qui-A 3)	A	France	Isolated element	7–11.5	?		
Le Piage 2	A	France	Isolated element	15 ± 36 months	?		Gambier, 2000
Les Rois A	A	France	Isolated element	10 ± 30 months	?	28,960 ± 210 (KIA 25247); 30,440 + 290/− 280 (KIA 25,248; 27,270 + 240/− 230 (KIA 25246)	Gambier, 2000; Ramirez Rozzi et al., 2009
Les Rois B	A	France	Isolated element	12 ± 30 months	?		
Les Rois E	A	France	Isolated element	9 ± 24 months	?		
Les Rois F	A	France	Isolated element	15 ± 36 months	?		
Mladeč 1	A	Czech Republic	Secondary deposition	16	?	31,190 + 400/− 390 (VERA-3073)	Frayer et al., 2006; Oliva, 2006; Svoboda, 2006; Teschler-Nicola, et al., 2006; Wild et al., 2006
Mladeč 2	A	Czech Republic	Secondary deposition	16	?	31,320 + 410/− 390 (VERA-3074)	
Mladeč 10	A	Czech Republic	Secondary deposition	18–19	?	34,160 + 520/– 490 (GRN- 26333); 34,930 + 520/− 49 (GRN 26334)	
Mladeč 47	A	Czech Republic	Secondary deposition	11–12	?		
Nazlet Khater 2 (NK 2)		Egypt	Primary deposition Single burial	20–29	M	33,000	Crevecoeur, 2012; Pinhasi and Semal, 2000; Vermeersch, 2002
Ostuni 1/Os1	G	Italy	Primary deposition With Os1b (foetus) within pelvic region	20	F	23,446 ± 107 (MAMS-11,449); 24,410 ± 320 (Gif-9247)	Chakroun et al., 2018; Fu et al., 2016; Giacobini, 2007; Nava et al., 2017; Ronchitelli et al., 2015
Paglicci 2	G	Italy	Primary deposition Burial	12–14	M	24,720 ± 420 (F-55)	Mussi, 2002; Ronchitelli et al., 2015
Paglicci 15	G	Italy	Primary deposition Burial	14	?		
Paglicci 25	G	Italy	Primary deposition Burial	18–25	F	23,470 ± 370 (F-57); 23,040 ± 380 (F-58)	
Pataud 1	G	France	Primary deposition Double burial (with Abri Pataud 2)	20–29	F	21,800 ± 90 (GrA-45013); 21,910 ± 90 (GrA-45133); 22,360 ± 90 (GrA-45132); 22,470 ± 90 (GrA-45016)	Chiotti et al., 2015; Henry-Gambier, 2008b; Nespoulet et al., 2006; Villotte et al., 2018
Pataud 3	G	France	Primary deposition Double burial (with Abri Pataud 4)	>20	F		
Pataud 5	G	France	Primary deposition Bones dispersed	>20	M?		
Paviland 1 (‘Red Lady’)	G	UK	Single burial Primary deposition	20–29	M	28,870 ± 180 (OxA-16412); 28,400 ± 320 (OxA-16502); 29,490 ± 210 (OxA-16413); 28,820 ± 340 (OxA-16503); 25,850 ± 280 (OxA-8025); 26,350 ± 550 (OxA-1815)	Henry-Gambier, 2008b; Jacobi and Higham, 2008
Pavlov 5	G	Czech Republic	Isolated element	21–25		26,170 ± 450 (GrN-20391)	Hillson et al., 2006; Svoboda et al., 2016; Trinkaus et al., 2010
Pavlov 16	G	Czech Republic		6–10			
Pavlov 17	G	Czech Republic		6–10			
Pavlov 18	G	Czech Republic		6–10			
Pavlov 22	G	Czech Republic		21–25			
Pavlov 23	G	Czech Republic		21–25			
Pavlov 29	G	Czech Republic		Early–mid second decade			
Pavlov 30	G	Czech Republic		Mid-second decade 8–16 years			
Pavlov 32	G	Czech Republic		5–12			
Předmostí 1	G	Czech Republic	Partial skeletons in mixed burial/pit	20–25	F	25,820 ± 170 (GrN-1286)	Klima, 1991; Svoboda, 2008; Ullrich, 1996
Předmostí 5	G	Czech Republic		12–16	F		
Předmostí 7	G	Czech Republic		12–14	?		
Předmostí 9	G	Czech Republic		20–25	M		
Předmostí 10	G	Czech Republic		20–25	F		
Předmostí 18	G	Czech Republic		20	M		
Předmostí 20	G	Czech Republic		9–10			
Předmostí 22	G	Czech Republic		9–11	?		
Předmostí 25	G	Czech Republic		<12	?		
Romito 2 (Romito dwarf)	EG	Italy	Double burial (with Romito 1)	17-20	M?	11,150 ± 150	Formicola, 2007; Giacobini, 2007
Sunghir 2	A/G	Russia	Multiple burial (with Sunghir 3 and 4)	12–13	M	23,830 ± 220 (OxA-9037); 27,210 ± 710 (AA-36474); 26,200 ± 640 (AA-36475); 30,100 ± 550 (OxX-2395-6)	Cowgill et al., 2015; Formicola and Buzhilova, 2004; Marom et al., 2012; Sikora et al., 2017; Trinkaus and Buzhilova, 2018; Trinkaus et al., 2014
Sunghir 3	A/G	Russia	Multiple burial (with Sunghir 2 and 4)	9–10	M	24,100 ± 240 (OxA-9038); 26,190 ± 640 (AA-36476); 30,000 ± 550 (OxX-2395-7)	
Tagliente/Tagliente 2	EG	Italy	Partially destroyed grave	20–29	M	13,190 ± 90 (OxA-10672)	Hedges et al., 1993; Gazzoni and Fontana, 2011; Giacobini, 2007
Vilhonneur 1	G	France	Single burial	15–25	M	27,010 ± 210 (Beta 216141); 26,690 ± 190 (Beta 216142)	Henry-Gambier et al., 2007

Chrono-cultural attribution: A, Aurignacian; B, Baradostian; G, Gravettian; EG, Epigravettian. Sex: F, Female; M, male.

Box 1. Upper Palaeolithic adolescents from Sunghir, Russia

In the Mid Upper Palaeolithic, ritualised burials emerge in Europe. Specifically, while there are only approximately 45 burials between 33,000 years and 22,000 years (Cal BP), they include double and triple burials of adults and subadults and are often associated with ochre and grave goods including elaborate items of personal adornment. In some cases, the individuals are placed side by side in a common shallow grave, with two individuals engaged (e.g. facing each other or somehow intertwined) and one individual disengaged. One of the most spectacular examples of a burial from this period is the double burial from Sunghir in Russia (Formicola, 2007; Sikora et al., 2017; Trinkaus & Buzhilova, 2018). Here, two adolescent boys – one (Sunghir 3) approximately 10 years of age and the other (Sunghir 2) closer to 12 years old – were buried head to head while a 35- to 45-year-old adult male (Sunghir 1) was buried adjacent to them. Next to the left humerus of Sunghir 2, archaeologists recovered the broken shaft of an adult femur (Sunghir 4) stuffed with ochre. The treatment and placement of the shaft suggests it was a grave good rather than a fourth internment. Other grave goods include hundreds of perforated arctic fox canines, ivory spears and pins, disc-shaped pendants, ivory and bone animal carvings including a horse head carved from a horse hyoid in the ‘contour découpé’ style. A total of 13,000 mammoth ivory beads were associated with the interred individuals with the youngest individual, Sunghir 3, having the greatest number – approximately, 5400 beads (Soffer, 1985). The beads, probably sewn on to clothing, are highly standardised and, remarkably, the ones associated with the adolescents are two-thirds the size of those associated with the adult male.

Both adolescents suffered from ‘repeated and pronounced periods of developmental stress, as is indicated by multiple dental enamel hypoplasias’ (Trinkaus & Buzhilova, 2018: 11). Furthermore, Sunghir 3 exhibits pronounced congenital bowing of both femora which would have been very noticeable during his life, although he apparently remained quite active based on the robusticity of muscle markings on his legs. While seemingly healthy, Sunghir 2 has very light masticatory muscle markings and almost no wear on his teeth as well as an unusual degree of alveolar prognathism. Trinkaus and Buzhilova (2018) believe their deaths may be related to their abnormalities. Sunghir 1 probably died very suddenly from a sharp cut to his neck (T1) which researchers suggest resulted from either a hunting accident or a social altercation (Trinkaus & Buzhilova, 2012, 2018). Sunghir 5 is an isolated adult cranium found above the burial of Sunghir 1 and Sunghir 7 is a possible adolescent femur found between the burial of Sunghir 1 and the double burial of Sunghir 2 and 3.

At Sunghir, we see that human remains received differential mortuary treatments – some remains are treated essentially as grave goods/cultural objects (Sunghir 4 and 5), while others appear to have been interred in the absence of archaeologically visible ritual (Sunghir 7) and still others are lavishly interred (Sunghir 1–3) (Trinkaus & Buzhilova, 2018). According to Soffer (1985), the beads associated with Sunghir 1–3 represent more than 2500 person hours of investment in the burials. Thus, it is particularly striking that the choice was made to take these beads out of circulation by burying them with the adolescents as they represent a substantial loss of labour. This ‘preference for the pathological’ is repeated at numerous sites across Europe (Trinkaus & Buzhilova, 2018: 17), leading researchers to suggest that Gravettian and Epigravettian peoples may have preferentially buried those members of their society who died ‘bad deaths’ or suffered from pathological conditions (Formicola, 2007; Pettitt, 2010; Sparacello et al., 2018).

Aside from skeletal remains, other direct biological indicators of individual people in the Upper Palaeolithic include finger-holes in clay, handprints, hand stencils, finger flutings and footprints recorded in multiple French and Spanish decorated caves including Aldène, Bedilhac, Chauvet, Cosquer, Font du Gaume, Fontanet, Gargas, Montespan, Niaux, Ojo Guareña, Pech Merle, Réseau Clastres and Tuc d’ Adoubert, and finger flutings from El Castillo, Gargas, Las Chimeneas and Rouffignac (Roveland, 2000; Clottes, 2013; Van Gelder, 2015a, b; Bahn, 2016). While it is possible to identify markings that are too small to have been made by adults, there is enough overlap between larger adolescents and smaller adults – particularly towards the end of puberty as stature increases – that this is not a reliable means of ‘seeing’ adolescents in the archaeological record (e.g. Sharpe & Van Gelder, 2006; Van Gelder, 2015a). The empirical data undermine assertions such as those made by Guthrie (2005: 126), that most handprints in Palaeolithic caves were made by adolescents; we simply cannot know that.

## Building a baseline for Upper Palaeolithic adolescence

As demonstrated in Box 1, the archaeological record offers direct data on the behaviour and lives of adolescents who lived during the European Upper Palaeolithic. However, these data are comparatively rare, and only provide ‘snapshots’ of the experience and roles of *some* adolescents in these societies. We should be particularly wary about extrapolating from the burial evidence to the wider adolescent experience. The 50–80 adolescent fossil individuals dated to the ~28,000-year span of the Upper Palaeolithic approximates 0.05 burials per human generation (although many of these fossils cluster chronologically to the Mid-Upper Palaeolithic ~35,000–25,000 years ago). Furthermore, many of these Upper Palaeolithic burials – including those from Sunghir discussed in Box 1 – are unusual, being recipients of elaborate burial rites (in the form of grave goods), and in many cases exhibiting pronounced developmental abnormalities that suggest they were in some way anomalous or exceptional (Wengrow and Graeber, 2015; Trinkaus, 2018; Sparacello et al., 2018).

The comparative lack of direct data need not deter us, however, from drawing some informed hypotheses about adolescents and the experience of adolescence in the Upper Palaeolithic. Here, we present our initial steps towards developing a baseline of the key features of adolescence in the Upper Palaeolithic. This baseline draws on two bodies of literature: (a) the developmental and biological data on adolescence as a uniquely human life stage described above; and (b) ethnographic accounts of adolescence in recent historical and extant hunter–gatherer societies. The fact that all European Upper Palaeolithic populations were *Homo sapiens* provides the justification for the former. As members of the same species, a principle of uniformitarianism (Howell, 1976; French & Chamberlain, in press) supports the position that the key developmental and biological features that define and characterise adolescence in recent human populations also apply to Pleistocene *Homo sapiens*.

In contrast, despite being a human universal, the variation in the experience and role of adolescence in human societies means that no equivalent principle of uniformitarianism is applicable from which to build a baseline for social adolescence in the Upper Palaeolithic. The best recent analogues for the experience and social perception of adolescence in the European Upper Palaeolithic, and for the roles and responsibilities of adolescents, are extant hunter–gatherer groups. We need to be careful when making such comparisons to avoid both replicating the present in the past and implying that these groups are anything other than present-day populations with their own unique histories and cultures (Wobst, 1978; Gould, 1980). Nonetheless, even at the level of a broad comparison, data from non-industrial societies are an automatic antidote to all-too-common assumptions that key stereotypes of recent Western adolescence – such as ‘teenage rebellion’ and identity crises – are universal (see, for example, Blakemore, 2018). Furthermore, our review of the key features of social adolescence among extant hunter–gatherers indicates that these are driven by features of these societies that are shared with Upper Palaeolithic hunter–gatherers, including small group sizes and low population densities.

### Social adolescence in recent hunter–gatherer societies

Much as is the case within archaeology, the study of adolescents in recent foraging societies is less common than the study of childhood (e.g. Montgomery, 2009; Konner, 2010; Meehan and Crittenden, 2016). Furthermore, adolescence is mostly either studied as part of childhood *sensu lato*, or otherwise undifferentiated. Many of the key features of hunter–gatherer adolescence do appear to have important precursors in childhood, and from an evolutionary perspective, developmental stages cannot be understood in isolation (Stambler, 2017: 25). Nonetheless, the adolescence of hunter–gatherers, like that of all human societies, is a distinct, marked stage of social life and development.

Hewlett and Hewlett (2012) have conducted the most comprehensive cross-cultural research on hunter–gatherer adolescence. In addition to characteristics of adolescence shared with other higher primates generally (e.g. an increase in sexual activity, an increase in time spent learning complex skills) and humans specifically (e.g. sexual division of labour, marriage), they identify multiple features of adolescence they consider unique to foragers (Table 2). We highlight a few of these here.

**Table 2.** Key characteristics of adolescence unique to hunter–gatherers (after Hewlett & Hewlett, 2012)

Characteristics of hunter–gatherer adolescence
Relatively high sexual freedom Long-distance exploration Autonomy and self-directed social learning Minimal and non-obligatory responsibility for subsistence and infant care Physical and emotional intimacy with parents and other adults Female initiation ceremonies Lack of adolescent identity crisis High levels of cultural energy, creativity and play

Many of the key features of forager adolescence are the result of demography (Hewlett & Hewlett, 2012: 96). While group composition is fluid (Dyble et al., 2015), the small size of forager camps (cross-cultural mean = ~25–30 people; Marlowe, 2005; Hill et al., 2011) means that hunter–gatherer children and adolescents are almost always physically close to a small number of people, within a network of widely dispersed groups living at low population densities. Furthermore, infant and child mortality rates are high in hunter–gatherer communities. Cross-culturally, ~43–49% of children die before the age of 15 (Hewlett, 1991; Marlowe, 2001, 2005; Volk & Atkinson, 2013). This combination of small group sizes and high childhood mortality means that, in contrast to many other societies, adolescents are rarely spending their time only with other adolescents; there are simply too few of them for this to occur. Adolescent socialisation and maturation thus occurs in an intergenerational context, alongside both younger children and adults, a factor that contributes notably to the ‘*physical and emotional intimacy with parents and other adults*’ that characterises hunter–gatherer adolescence.

This physical and emotional intimacy that characterises forager adolescence has its roots in childhood. Combined with two other important features of hunter–gatherer childhood – the development of individual autonomy and a strong sense of self and belonging caused by homogeneity of world view (Hewlett, 2017) – this intimacy manifests in one of the most notable features of forager adolescence: *the lack of the teenage ‘rebellion’ or identity crisis* that is considered the hallmark of social adolescence in many other societies (see, for example, Blakemore, 2018).

An example of social and economic changes caused by contact with twentieth-century Western cultures demonstrates the importance of hunter–gatherer demography and worldview in shaping the experience of social adolescence. Condon (1990) describes how the behaviour of teenagers of the Canadian Inuit community of Holman Island altered drastically following interventions by the federal government. Some of the key changes were demographic. Centralisation of settlements and accompanying population growth meant that, for the first time, there was a sizable peer group of adolescents. In combination with the beginnings of formal schooling, teenagers began to spend more of their time with their same-aged peers, rather than in mixed-age groups with their parents and other Inuit adults. Schooling also exposed the teenagers to other value systems and non-Inuit economic and social roles to aspire to, roles and employment that were nonetheless very difficult for Inuit youths to obtain. The concomitant increased influence of peers and decreased parental and elder influence, combined with their exposure to new values and social expectations that contrasted with those of Inuit society, led to a stark increase in troubling adolescent behaviour including the drinking of alcohol and violence. Notable changes were also witnessed in other domains, including the selection of marriage partners (now less influenced by parental choice) and a delay in marriage age, which slowed down the social transition from childhood (*sensu lato*) to adulthood in Inuit society.

Adolescence among hunter–gatherers is a time during which individuals seek sexual and potential marriage partners, a feature they share with teenagers in other societies and well as higher primates who have just attained reproductive maturity. The search for marriage and sexual partners, combined with the low population densities of foragers – the cross-cultural mean of which is 0.25 persons/km^2^ (Marlowe, 2005) – is responsible for another of the characteristic features of hunter–gatherer adolescence: *long distance exploration*. Among foragers, there is a significant inverse correlation between mating distance and population density; the lower the regional population density is, the greater the distance travelled to find a suitable partner (MacDonald & Hewlett, 1999). Furthermore, it is during adolescence or young adulthood that the greatest distances are travelled, and men usually travel further than women. This latter finding accords with sexual selection theory that proposes that the sex with the higher potential reproductive rate will compete more strongly for access to potential partners (*ibid*.). While it is hard to state with certainty the reason for specific instances of long-distance travel (see for example, Hagino & Yamaucho, 2014), there is no doubt that this is a frequent occurrence among teenage hunter–gatherers. Ethnographers commonly note the high levels of absence of adolescent boys in hunter–gatherer camps (e.g. Hagino & Yamaucho, 2014; Milner et al., 2014). Evolutionarily, there may have been selection for navigation skills in adolescent males. In a study of male and female West Point cadets, Munion et al. (2019: 1933) note that ‘traveling longer distances without changing course, pausing less, and fewer returns to previously visited locations were significantly related to the ability to locate the correct target … [and] the significant relationship between gender and navigational success is fully accounted for by men and women producing different wayfinding behaviors, which in turn predict differences in navigational success’.

Finally, the shifts in sleeping patterns that occur during adolescence are of particularly importance within forager communities. Samson et al. (2017) studied the relationship between chronotype variation (the times of day that a person feels the most alert or the greatest need for sleep) and group sleeping among the Hadza, a people who practice a foraging lifestyle in Northern Tanzania. Over a 20-day period, they found that there were only 18 one-minute episodes in which everyone was simultaneously asleep, while 99.8% of the time one person or more was awake. Chronotype varied by age only and not by sex, co-sleeping, nursing or study day. They argue that variation in age helps to generate variation in chronotype which facilitates sentinel-like behaviour which evolutionarily would have been key to group survival (e.g. for detection of danger from predators, other humans and the environment). This variation in sleeping patterns is particularly important for the small groups in which hunter–gatherers usually live and camp (12 or fewer). In models where they controlled for chronotype variation (i.e. they assumed that all members of a group fell asleep and woke at roughly the same time), groups had to be significantly larger for individual variation (e.g. someone wakes because they are too hot, have a nightmare, are hungry, need to urinate) to produce the same ‘sentinel effect’ (Samson et al., 2017). Thus, as teens enter puberty, they begin to play an increasingly important role in overall group safety.

### Adolescence in the European Upper Palaeolithic

The features listed in Table 2 are particularly characteristic of mobile, immediate-return hunter–gatherers – those who lack storage, are generally egalitarian, have a culture of extensive giving and sharing, and live in small (<30 individuals) groups at low population densities (Hewlett & Hewlett, 2012). Along the simplified spectrum from ‘simple’, egalitarian, immediate-return hunter–gatherers to ‘complex’, hierarchical, delayed-return hunter–gatherers (Woodburn, 1982; Keeley, 1988), European Upper Palaeolithic hunter–gatherers – with their evidence of substantial storage, some semi-permanent dwelling structures and the presence of social hierarchies (e.g. Soffer, 1985; Fišaková, 2013; Iakovleva, 2015; cf. Wengrow and Graeber, 2015) – fall closer to the latter. Nonetheless, we still consider the features listed in Table 2 to be highly relevant to our understanding of the experience of social adolescence in the Upper Palaeolithic, with variability in adolescence resulting from different foraging adaptations being of a degree rather than of a kind.

The relationship between demography and the key characteristics of forager adolescence provides the main support for this assertion. The demography of European Upper Palaeolithic foragers is hard to infer from archaeological and paleoanthropological data (French, 2016), but in general terms resembles that of recent foragers. The European Upper Palaeolithic metapopulation fluctuated across its ~28,000-year span, with absolute estimates ranging from ~1,500 to 11,000 people in Western and Central Europe (Maier, 2017). Outside of some key areas (e.g. southwestern France, the Danube basin) regional populations were small and lived at low densities in small, mobile groups (Bocquet-Appel et al., 2005; French, 2015; French & Collins, 2015; Kretschmer, 2015; Maier & Zimmerman, 2017; Schmidt & Zimmerman, 2019). Estimates of Upper Palaeolithic fertility and mortality rates come with many caveats, but similarly indicate high rates of both adult and child mortality (Trinkaus, 2011). Accordingly, we propose that the behaviours described above, and listed in Table 2 are an appropriate ‘baseline’ for social adolescence in the Late Pleistocene.

While the uniformitarian principle allows us to draw broad parallels between past and present in the developmental and biological aspects of adolescence, the aforementioned plasticity in the onset, pace and duration of puberty – plasticity that is strongly influenced by social and environmental factors – should prompt us to ask if and how hunter–gatherer adaptations influence biological adolescence. The age of onset of female adolescence has received particular attention, with an increasingly early age of puberty (marked by the onset of menarche) recorded in industrialised countries over the last 100 years, a trend that has been linked to, among other factors, energy balance and nutrition (Garn, 1987). The link between onset of menarche and energy probably explains the contrasts seen between hunter–gatherer and modern industrial societies in this variable. Jones and Lopez (2006: 47) report an average age at menarche for hunter–gatherer girls of 16.1 years (compared with 12.1 years for girls in the USA), 19.5 years for age at first birth (26 years in the USA) and an interval of 3.4 years between menarche and first pregnancy (13.5 years in the USA). While the latter is heavily influenced in modern industrial populations by age at marriage, among hunter–gatherers it is more likely to be the result of adolescent subfecundity (the period of up to several years of irregular/anovulatory cycles that follows the onset of menstruation, which may be compounded by low calorie intake), as marriage may precede menarche (Hochberg & Konner, 2020: 7). If, as is the case among the !Kung, the adolescent life stage (for women) is defined as the period between the onset of menarche and the birth of the first child, ‘adolescents’ are those between approximately 16 and 21 years of age (Howell, 2010). Among !Kung men the period of adolescence is longer, lasting on average from 16 to 27, and similarly ending with the birth of the first child (*ibid*.).

Direct data on the onset and duration of puberty and adolescence among Upper Palaeolithic children is lacking. To the best of our knowledge, the aforementioned skeletal maturation indicators of puberty of Lewis et al. (2016; Shapland and Lewis, 2013, 2014) have not been applied to any Upper Palaeolithic individuals. Some European Upper Palaeolithic populations appear to have been in reasonably good health and nutritional condition (e.g. those of the Mid-Upper Palaeolithic or Gravettian; Holt & Formicola, 2008; Formicola & Holt, 2015), which may have contributed to lowering the age of onset of puberty. The Ostuni 1 woman, who was heavily pregnant at the time of her death at age 20 years (Nava et al., 2017), provides the only firm evidence for age at pregnancy in the European Upper Palaeolithic, giving a *terminus ante quem* for the end of the period of adolescent subfecundity for this individual (although it is unknown whether this was her first pregnancy). If the end of adolescence was defined in similar ways in past and present hunter–gatherer societies (i.e. by the age at first birth), then it is likely that female Upper Palaeolithic hunter–gatherer adolescence similarly lasted until the late teens/early twenties, given the role of biology in determining this variable; adolescence may have lasted even longer for men. Further study of adolescents in the European Upper Palaeolithic should focus on those older teens identified in Table 2, rather than the young teens (~10 years) (cf. Gluckman & Hanson, 2006).

## Hunter–gatherer adolescence and innovation

Hewlett and Hewlett (2012) further identify *high levels of cultural energy, creativity and play* as characteristic of hunter–gatherer adolescence. Two intersecting features likely drive this: (a) the increased cognitive abilities and other developmental features described earlier that make the human adolescent stage one in which creativity, flexibility, exploration and risk taking come to the forefront; and (b) the relatively large amount of free time that adolescent foragers have compared to adult foragers as a result of one other characteristic feature of hunter–gatherer adolescence – their *minimal and non-obligatory responsibility to participate in subsistence tasks and baby care*.

Creativity and play are fundamental to the development of new ideas and innovations (Nowell, 2016; Riede et al., 2018). Accordingly, we might ask whether there are any documented relationships between this unusually high rate of creativity and play exhibited by foragers during their teenage years and the invention and spread of new ideas and practices. Assuming that social norms, behaviours and traditions are taught to individuals within their culture, the study of innovation is linked with that of social learning (Terashima & Hewlett, 2016), a practice which, despite a recent resurgence in research, is still comparatively understudied and poorly understood among hunter–gatherers. Direct data on patterns and processes of innovation are rarer still. Nonetheless, some cross-cultural data are available from which to draw preliminary conclusions about how adolescent hunter–gatherers learn social and cultural practices and their contribution to innovations in forager societies.

With adolescence, the mode of hunter–gatherer social learning and cultural transmission changes. During their teenage years, foragers increasingly learn social norms and subsistence skills via oblique transmission (i.e. from non-parental adults), superseding the vertical transmission (i.e. from parent-to-child) or horizontal (i.e. from peers of the same generation) transmission that prevailed during childhood (Dira & Hewlett, 2016; Garfield et al., 2016). Forager demography is, once again, partly responsible: given comparatively high adult mortality and divorce rates, many teenagers will not have both parents. However, adolescence is an important time for skill learning, and the frequency of teaching is often higher than in earlier stages of childhood, as more complex and culturally valued skills are learned (Boyette & Hewlett, 2018). Two observations by Lew-Levy et al. (2017) are particularly noteworthy here. The results of their cross-cultural study of the social learning of forager subsistence skills show that adolescents are not innovators in this domain but instead preferentially seek out adults identified as innovators from whom to learn. They are also the main group to whom these innovations are transmitted. Adolescents are therefore not the primary innovators but are the primary recipients of innovations. Furthermore, Lew-Levy et al. (2017) suggest that these innovations are often learned and subsequently used by adolescents to aid in their search for suitable marriage and sexual partners – they are integral to the *long-distance exploration* that characterises the life of teenage foragers.

Based on the patterns seen among recent hunter–gatherers and the experience and roles of adolescents in these societies, we hypothesise that adolescents – with their high mobility, high creative energy and need to meet and interact with new groups in their search for potential marriage partners – played a key role in information sharing and the spread, and possibly also the origin (Riede et al., 2018), of innovations during the Late Pleistocene. The Upper Palaeolithic in Europe is characterised by the emergence of geographically extensive social networks, large-scale group aggregations, increases in innovations and diversification in material culture, in terms of both the range of materials used and their stylistic attributions (Langley et al., 2008; d’Errico & Stringer, 2011; Pettitt, 2014). Two other features also differentiate the Upper stage of the European Palaeolithic from its earlier stages: the habitation of the continent by *Homo sapiens* and a notable increase in continental population size and density (Mellars & French, 2011; Schmidt & Zimmerman, 2019). Both of these features have been evoked as explanations for the social and cultural changes seen with the European Upper Palaeolithic (e.g. Mithen, 1996; Shennan, 2001; Mellars, 2005; Powell et al., 2009), possibly in some kind of feedback loop (i.e. some biological difference between *Homo sapiens* and their Neanderthal predecessors in Europe – usually presumed to be related to cognitive abilities – facilitated population growth). However, a more pertinent biological difference between Neanderthals and *Homo sapiens* in terms of the evidence for the more extensive social networks, large-scale group aggregations, diversification of material culture and increase in innovations among the latter, is their possible slower pace of development, resulting in a pronounced and extended period of adolescence (both biological and social; Smith et al., 2007, 2009; Thompson & Nelson, 2011; Nowell, 2016, cf. Rosas et al., 2017). An extended developmental and social period of creativity and social learning with additional years to practice, transmit, modify and innovate upon aspects of their culture, within a fairly protected environment combined with high mobility (before reproduction and child-rearing occurs), would have contributed to strengthening existing social bonds and networks and creating new ones, as well as to the spread of ideas and innovations (see also Nowell, 2016). Accordingly, we hypothesise a key role of adolescence and adolescents in Late Pleistocene social and cultural patterns and changes, both in Upper Palaeolithic Europe and in comparable contexts elsewhere.

## Conclusion

Adolescence is unique to the human life history course. Driven by important biological and cognitive developments that occur with the onset of puberty, adolescence is universally recognised across human societies as a distinct life stage during which teenagers begin to prepare for adult life. The specifics of this social adolescence, including the roles and responsibilities of adolescents, their behavioural norms and relationships with both adults and younger children, vary cross-culturally. While less widely studied than childhood in prehistory, a consideration of the experience of adolescence is essential to a fuller understanding of all past societies.

Here, we have constructed a baseline for the study of adolescent lifeways in the European Upper Palaeolithic, drawing on bioarchaeological evidence, human developmental universals and social adolescence in forager societies. Recent hunter–gatherers are not direct and unproblematic analogues for Palaeolithic hunter–gatherers. Nonetheless, both past and prehistoric forager societies share many demographic similarities, including small group size, low population density and high mortality. The relationship between demography and the key characteristics of forager adolescence in the present provide the main support for our assertion that the key features characteristic of social adolescence among recent foragers, listed in Table 2, would also have been part of the experience of adolescence in the Upper Palaeolithic. We use this baseline for adolescence in the Upper Palaeolithic to argue that teenagers played an important, and heretofore largely unrecognised, role in the spread of the new ideas and innovations that characterised this period, as well as driving other key features of the archaeological record of Late Pleistocene hunter–gatherers, including increased long-distance connectivity and periodic large-scale aggregations.

Gender is one variable that we have not focused on here, but that demands further attention in this context. In the Upper Palaeolithic, as elsewhere, adolescence would have been experienced differently – both biological and socially – by girls and boys. The study of male adolescence dominates the literature (Feixa, 2011: 1636) but should not be mistaken for the universal experience. One key characteristic of adolescence among recent foragers that is particularly noteworthy here is the regular practice of female adolescence initiation ceremonies (Hewlett & Hewlett, 2012). While the empirical basis is shaky, if, as Guthrie (2005) suggests, handprints and paintings on Upper Palaeolithic cave walls were linked to adolescent rituals and initiation ceremonies, the recent forager data suggest that we might want to consider that the participants were teenage girls, not the teenage boys that Guthrie envisions. Future planned work, exploring in greater detail the bioarchaeological evidence for Upper Palaeolithic adolescents presented in Table 1, will contribute to the further study of adolescence in the Late Pleistocene, and to a richer understanding of Upper Palaeolithic lifeways more broadly.

## Data Availability

The data used in this study are published here in part and will be published in full in 18 months. They can be accessed by contacting the corresponding author.
